# Evaluation of the Updated Diagnostic Criteria for Paraneoplastic Neurologic Syndromes in China

**DOI:** 10.3389/fimmu.2022.790400

**Published:** 2022-01-31

**Authors:** Meng-Ting Cai, Song Qiao, Qi-Lun Lai, Yang Zheng, Fan Yang, Gao-Li Fang, Chun-Hong Shen, Yin-Xi Zhang, Mei-Ping Ding

**Affiliations:** ^1^ Department of Neurology, Second Affiliated Hospital, School of Medicine, Zhejiang University, Hangzhou, China; ^2^ Department of Neurology, Zhejiang Hospital, Hangzhou, China; ^3^ Department of Neurology, The First Affiliated Hospital of Zhejiang Chinese Medical University, Hangzhou, China; ^4^ Department of Neurology, People’s Hospital of Anyang City, Anyang, China; ^5^ Department of Neurology, Zhejiang Chinese Medicine and Western Medicine Integrated Hospital, Hangzhou, China

**Keywords:** paraneoplastic neurologic syndrome, updated diagnostic criteria, clinical phenotype, antibody, cancer

## Abstract

**Background:**

Recently, the paraneoplastic neurologic syndrome (PNS) diagnostic criteria have received a major update with a new score system over the past 16 years. We aimed to evaluate the diagnostic performance and clinical utility in China.

**Methods:**

An eligible cohort of 113 Chinese patients diagnosed with PNSs from the Second Affiliated Hospital School of Medicine Zhejiang University and published data were enrolled retrospectively. Data including clinical phenotype, antibody type, the presence of cancer, and duration of follow-up were reviewed and re-evaluated to classify the diagnostic levels for the 2004 and 2021 PNS criteria. The performances of these 2 criteria were compared. The critical parameters of antibody and cancer for the updated criteria were further explored.

**Results:**

The cohort consisted of 69 males and 44 females with a median age of 60 years. Limbic encephalitis (23, 20.4%), anti-Hu antibody (32, 28.3%), and small-cell lung cancer (32, 28.3%) were the most common clinical phenotype, detected antibody, and concomitant cancer, respectively. A total of 97 (85.8%) patients were diagnosed with definite PNS according to the 2004 criteria: only 42.3% (41/97) fulfilled the 2021 criteria, while the remaining 40, 14, and 2 re-diagnosed with probable PNS, possible PNS, and non-PNS. The requirement of cancers consistent with antibody and phenotype increased the specificity and thus greatly enhanced the accuracy of the 2021 criteria.

**Conclusion:**

The updated criteria for PNS emphasized the consistency between cancer phenotype and antibody and showed a better diagnostic value. A better diagnostic yield could benefit disease management.

## Introduction

Paraneoplastic neurologic syndromes (PNSs) are a group of neurological disorders in association with cancer and have an immune-mediated pathogenesis that is supported by the frequent presence of specific neuronal antibodies (1, 2). Their diverse and complicated presentations with involvement of the nervous system had various mimics, including infections, autoimmune non-paraneoplastic diseases, tumors, neurodegenerative disorders, and toxic/metabolic disturbances (3, 4). Even though the detection of specific antibodies and cancers provided great favor for identification, a certain proportion of patients might still be difficult to diagnose. Recent population-based studies suggested an increasing incidence of PNS over time with a range of 2–10 per million person-years, and it developed in 1 in every 334 patients with cancers (5–8). Apart from the increased awareness and improved detection techniques, updated criteria were needed to increase the diagnostic rate, especially for those with new antibodies.

Recently, the PNS-Care panel updated the diagnostic criteria for PNS on the basis of the new phenotypes and antibodies since the proposal of the first criteria in 2004 (9, 10). A new clinical scoring system (PNS-Care Score) was developed to increase diagnostic accuracy in complicated clinical scenarios. The diagnostic certainty was divided into 3 levels (possible, probable, and definite PNS) according to the coherence between clinical phenotype, antibody, and cancer. And 3 new risk categories (high-risk, intermediate-risk, and low-risk phenotypes) were created to stratify antibodies and their associated syndromes with more precise terminologies (11). As suggested, the new criteria provided a broad, comprehensive approach for unambiguous diagnostic certainty, yet patients with negative neuronal antibodies might be underestimated (10, 11). However, no study has evaluated the application of the new criteria to date. We therefore aimed to evaluate the validity of the updated diagnostic criteria for PNSs in a cohort of Chinese patients and to compare the diagnostic performance with the 2004 PNS criteria.

## Methods

### Study Design

**Figure 1:**
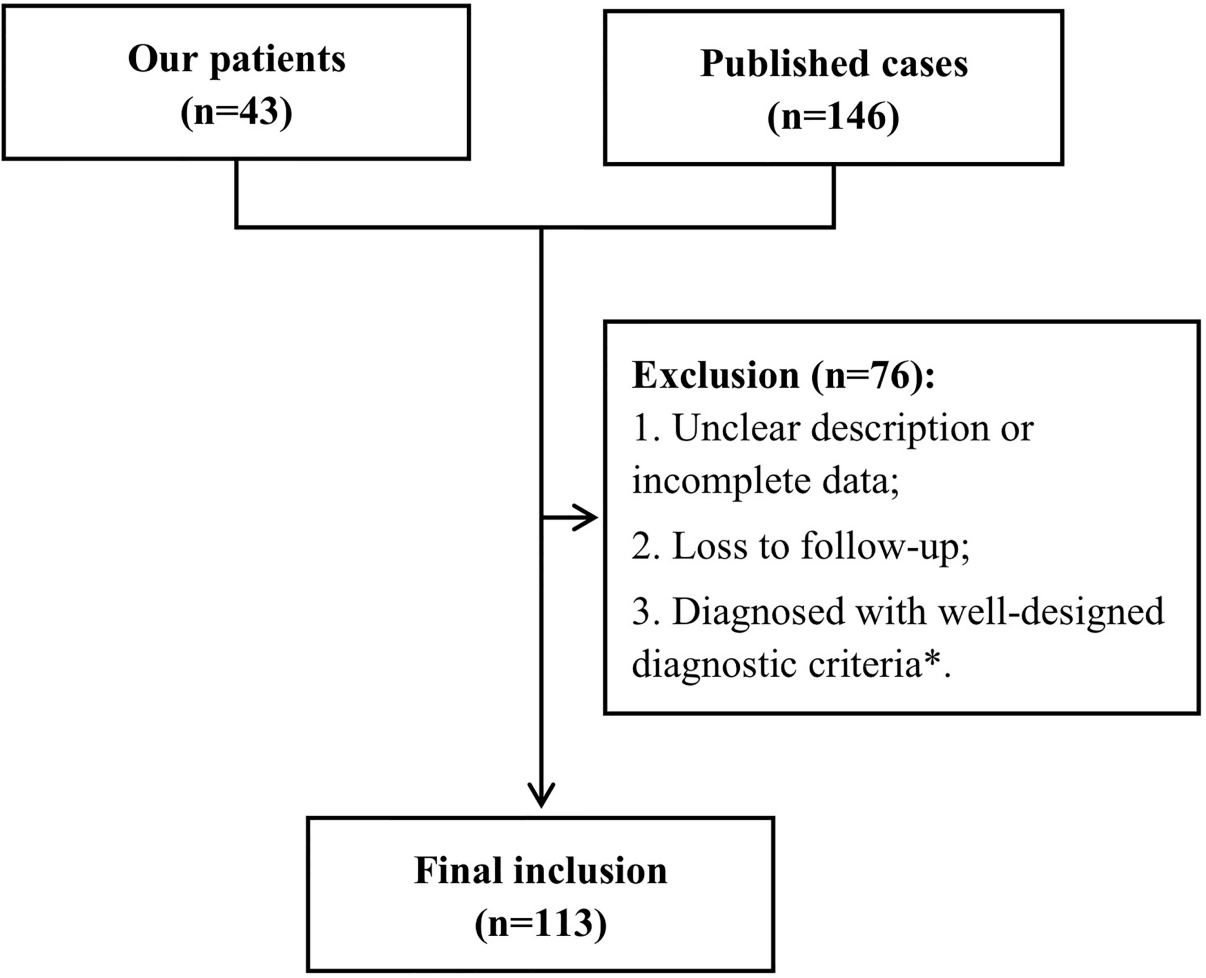
Flowchart. *Diseases occurred in association with cancer but had well-designed diagnostic criteria (10), including inflammatory myopathies (dermatomyositis, polymyositis, and necrotizing myopathies), myasthenia gravis, and polyneuropathies associated with monoclonal gammopathies, and paraneoplastic retinopathy, optic neuritis, and cochlear vestibulopathy.

We retrospectively collected 189 cases with a diagnosis of possible or definite PNS by the 2004 criteria (9) from 2 origins. Forty-three patients with a follow-up over 2 years were enrolled from the Second Affiliated Hospital School of Medicine Zhejiang University between January 2016 and June 2019. Others were from PubMed, Web of Science, and Embase by 2 independent investigators from January 2004 to April 2021 (12–45). Relative terms (including variations) of “paraneoplastic neurologic syndrome”, “paraneoplastic neurological syndrome”, and “paraneoplastic syndrome, nervous system” were used for search with restriction of affiliation in China. We retrieved all relevant articles and searched their reference tables to identify as many additional studies as possible. Only full articles in English and Chinese were included in the review; publications in abstract form were not considered. Detailed clinical data were collected, including the clinical phenotypes, antibodies (with detection methods), associated tumors, follow-up duration, demographic characteristics, other clinical or paraclinical results, and prognosis. Among them, cases with missing data were removed. Furthermore, patients with the following diagnoses were also excluded according to the updated criteria (10): inflammatory myopathies (such as dermatomyositis, polymyositis, and necrotizing myopathies), myasthenia gravis, polyneuropathies associated with monoclonal gammopathies, and paraneoplastic retinopathy, optic neuritis, and cochlear vestibulopathy. The flowchart is presented in **Figure 1**. Our study was approved by the ethics committee of the Second Affiliated Hospital School of Medicine Zhejiang University.

### Evaluation of the 2 Criteria

The 2004 criteria classified the diagnosis as definite and possible levels mainly according to the presence of well-characterized antibodies and/or cancers (**Supplementary Table 1**) (9). And the 2021 criteria were divided into 3 diagnostic certainties with 3 parameters of clinical level, laboratory level, and cancer. The range scores corresponded to definite (≥8), probable (6–7), and possible (4–5) diagnoses of PNSs, respectively, as a score ≤ 3 was considered as non-PNS (**Supplementary Table 2**) (10). The initial data of all patients were reviewed and re-evaluated using the 2004 and 2021 criteria based on the current time by 2 neurologists independently. A final consensus was achieved after discussion in any discrepant cases. We calculated the diagnostic values of the 2 criteria and compared their performance in the setting of different tumor and antibody conditions.

### Statistical Analyses

All analyses were performed with R software. The results were presented as median [interquartile range (IQR)] or number (%). Chi-square test and Mann–Whitney U test were used to calculate the difference between different groups. The statistical significance was adopted at p < 0.05. We calculated the number of true positives (TP; presence of antibody/cancer in patients with definite PNS), true negatives (TN; absence of antibody/cancer in patients without definite PNS), false positives (FP; presence of antibody/cancer in patients without definite PNS), and false negatives (FN; absence of antibody/cancer in patients with definite PNS). Significant differences were determined with 95% confidence intervals. The sensitivity, specificity, positive predictive value, negative predictive value, and accuracy were calculated.

## Results

### Clinical Manifestations

**Figure 2:**
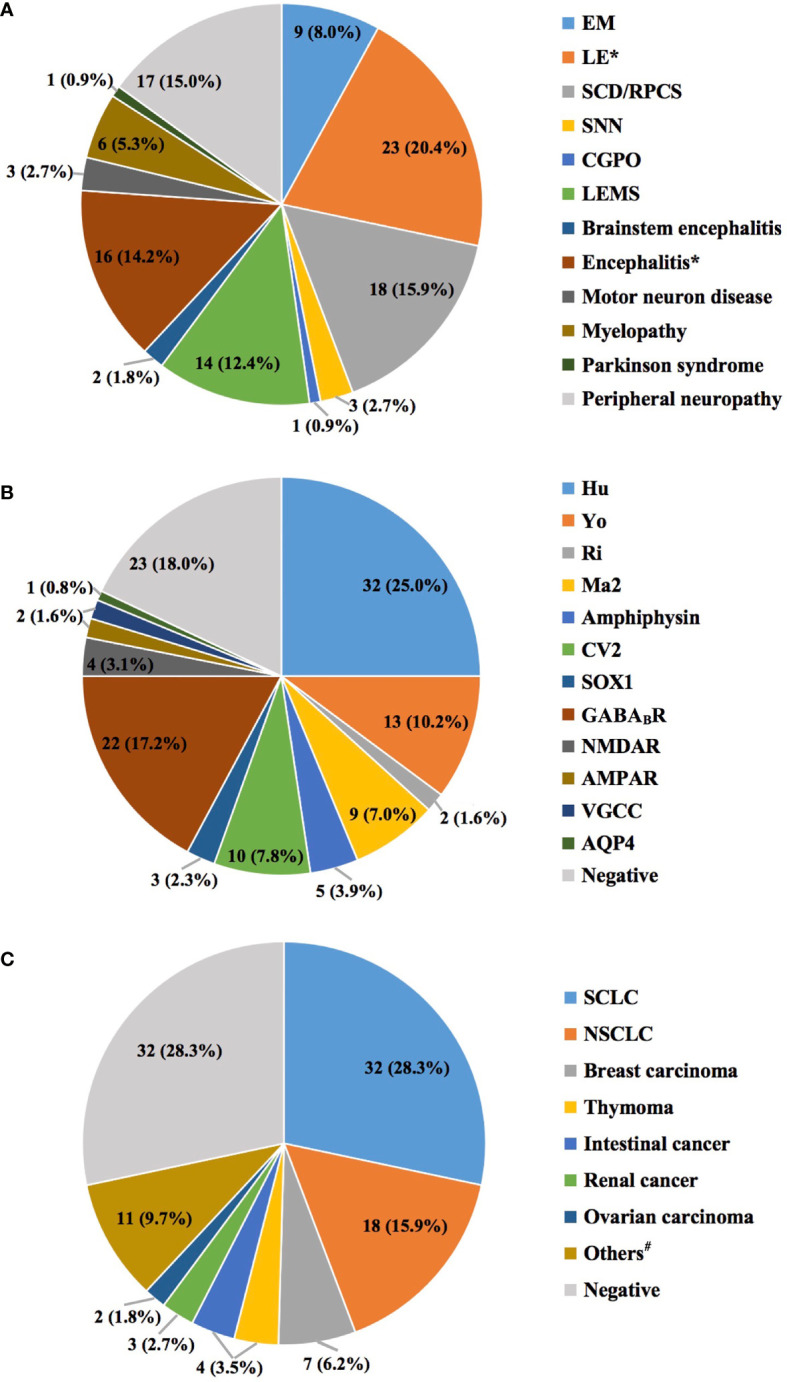
The distributions of clinical phenotypes **(A)**, antibody types **(B)**, and cancers **(C)**. The total number of clinical phenotypes and cancers was 113, compared with 128 antibodies. The former 2 features were unique in each patient, while coexisting antibodies were found in 14 patients (13 with 2 antibodies, and 1 with 3 antibodies). *Only 23 of the 35 patients initially diagnosed with LE fulfilled the 2016 *Lancet* criteria (46), and the rest were classified as encephalitis. ^#^Eleven types of cancers for each patient, including testicular cancer, cervical carcinoma, spinal cord tumor, palatal squamous epithelial carcinoma, lymphoepithelial carcinoma, mucosa-associated lymphoid tissue lymphoma, multiple myeloma, non-Hodgkin lymphoma, bladder cancer, ganglioneuroma, and gastric carcinoma. AMPAR, α-amino-3-hydroxy-5-methyl-4-isoxazolepropionic acid receptor; AQP4, aquaporin 4; CGPO, chronic gastrointestinal pseudo-obstruction; EM, encephalomyelitis; GABA_B_R, gamma-aminobutyric acid-B receptor; LE, limbic encephalitis; LEMS, Lambert–Eaton myasthenic syndrome; NMDAR, *N*-methyl-d-aspartate receptor; NSCLC, non-small-cell lung cancer; RPCS, rapidly progressive cerebellar syndrome; SCLC, small-cell lung cancer; SNN, sensory neuronopathy; VGCC, voltage-gated calcium channel.

A total of 113 patients from 24 centers in 11 provinces across China were eventually included in the study, with the largest proportion of our cohort in Zhejiang Province (43, 38.1%). They were between the ages of 29 and 83 (median 60.0, IQR = 53.0–65.0) and were mainly males (61.1%). Among the clinical phenotypes, limbic encephalitis (LE) was the most frequent (23, 20.4%), followed by subacute cerebellar degeneration (SCD)/rapidly progressive cerebellar syndrome (RPCS) (18, 15.9%) and Lambert–Eaton myasthenic syndrome (LEMS) (14, 12.4%) (**Figure 2A**). Referring to the positively associated antibodies, the anti-Hu antibody was the most frequent (32/90, 35.6%), followed by anti-gamma-aminobutyric acid-b receptor (GABA_B_R) antibody (22/90, 24.4%) and anti-CV2 antibody (13/90, 14.4%) (**Figure 2B**). There were 14 patients with at least 2 coexisting antibodies. Interestingly, 12 of them were positive for anti-Hu antibodies, with a frequent combination of anti-GABA_B_R antibodies (8/12, 66.7%) and even triple antibodies with anti-*N*-methyl-d-aspartate receptor (NMDAR) antibodies (2/12, 16.7%). Coexistence of anti-Yo and anti-CV2 antibodies was found in 2 patients, as other combinations of anti-Hu plus anti-Yo/SOX1/Ri antibodies and anti-Yo plus anti-voltage-gated calcium channel (anti-VGCC) antibodies were each found in 1 patient. More than 20 types of tumors were found in 81 patients, most of which were lung cancers, including small-cell lung cancer (SCLC) (32/81, 39.5%) and non-SCLC (NSCLC) (18/81, 22.2%) (**Figure 2C**). Among them, there were 42 patients receiving oncotherapies. Among 81 patients with cancers, 27 died, 6 worsened, and 28 obtained remission, as compared with those without cancers of 7, 4, and 19, respectively. **Table 1** exhibits the details.

**Table 1:** Summary of patients with PNSs.

	Overall (n = 113)
**Age at onset, years, median (IQR)**	60.0 (53.0–65.0)
**Males, n (%)**	69 (61.1)
**Antibodies, n (%)**	
Positive	90 (79.6)
Negative	23 (20.4)
Coexisting antibodies	14 (12.4)
**Cancers, n (%)**	
Detected	81 (71.7)
Negative	32 (28.3)
**Oncotherapies, n (%)**	42 (37.2)
Monotherapy	
Surgery	15 (13.3)
Chemotherapy	14 (12.4)
Radiotherapy	1 (0.9)
Combined therapies*	12 (10.6)
Unknown	18 (15.9)
**First-line immunotherapies, n (%)**	
Steroids monotherapy	19 (16.8)
IVIG monotherapy	10 (8.9)
Steroids combined with IVIG	12 (10.6)
**Outcomes, n (%)**	
Death	34 (30.1)
Deterioration	10 (8.8)
Stabilization	15 (13.3)
Remission	47 (41.6)
Unknown	7 (6.2)

IQR, interquartile range; IVIG, intravenous immunoglobulin; PNS, paraneoplastic neurologic syndrome.

*Any combination of surgery, chemotherapy, and radiotherapy.

The characteristics of patients with specific antibodies are shown in **Supplementary Table 3**. The patients with anti-SOX1 antibody were the oldest (median = 69.0 years, IQR = 65.0–72.0), with the highest proportion of males (100%). LE was the most frequent syndrome in various antibodies including anti-amphiphysin, anti-GABA_B_R, anti-NMDAR, and anti-α-amino-3-hydroxy-5-methyl-4-isoxazolepropionic acid receptor (AMPAR) antibodies, corresponding to SCD/RPCS in anti-Yo and anti-CV2 antibodies, LEMS in anti-SOX1 and anti-VGCC antibodies, and peripheral neuropathy in anti-CV2 and anti-Ma2 antibodies. The concomitant cancers varied among each antibody, yet patients with high-risk antibodies of anti-Yo, anti-Ma2, and anti-Ri antibodies showed lower involvement rate of 53.8%, 55.6%, and 50.0%, respectively. NSCLC was found to be the most frequent among anti-Hu (12/32, 37.5%) and anti-GABA_B_R (6/22, 27.3%) antibodies, while SCLC was the most common among anti-CV2 (3/10, 30.0%) and anti-amphiphysin antibodies (2/5, 40%). Regarding the outcomes, patients with anti-Hu and anti-SOX1 antibodies showed higher mortality (40.6%, 66.7%), while more patients with anti-Yo and anti-CV2 obtained remission (61.5%, 50.0%).

According to the updated criteria, SCLC, NSCLC, breast carcinoma, ovarian carcinoma, and lymphomas were highly relevant with PNSs irrespective of the antibody status. As shown in **Supplementary Table 4**, patients with SCLC presented more frequently with LE and LEMS (both 8/32, 25.0%) and combined mostly with anti-Hu antibody (5/32, 27.8%). The patients with NSCLC had more encephalitis (5/18, 27.8%) and anti-Hu antibody (12/18, 66.7%). By contrast, only a few patients were found to have breast carcinoma (n = 7), ovarian carcinoma (n = 2), and lymphomas (n = 1). However, the small number of patients in each cancer category prevented us from reaching a definite conclusion.

### Evaluation of Patients With the 2 Criteria

**Figure 3:**
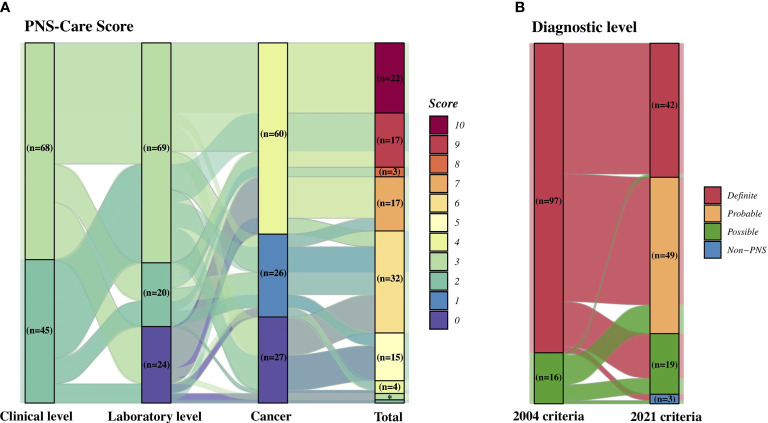
Diagnostic performance of the 2 criteria. **(A)** Number of patients corresponding to PNS-Care score. **(B)** Comparison of diagnostic levels between the 2004 and 2021 PNS criteria. PNS, paraneoplastic neurologic syndrome. *There were 2 patients with a total score of 3 and 1 patient with a score of 2.

The PNS criteria are mainly based on 3 aspects consisting of a neurologic syndrome, antibody, and cancer, even though the definitions of each factor were somewhere modified and updated. As shown in **Table 2**, each classification and the number of corresponding patients were rather different. In detail, the classical syndromes in the 2004 criteria were divided into high-risk (68/80, 85.0%) and intermediate-risk (12/80, 15.0%) phenotypes in the 2021 criteria, as the latter 12 patients with a previous diagnosis of LE failed to fulfill the 2016 *Lancet* criteria by Graus et al. (46). The high-risk antibodies consisted of the well-characterized antibodies by the 2004 criteria (67, 59.3%) and the new anti-SOX1 antibody (2, 1.8%). Yet no partially characterized antibody was found. The intermediate-risk antibody group included 20 patients with anti-GABA_B_R, anti-AMPAR, anti-NMDAR, and anti-VGCC antibodies. The low-risk antibody [anti-aquaporin 4 (anti-AQP4) antibody] was only found in 1 patient. Importantly, the updated PNS criteria had more specific and stricter classifications for clinical, laboratory, and cancer parameters. Reflected by PNS-Care Score system (**Figure 3A**), the median scores of the parameters in this cohort were 3.0 (IQR = 2.0–3.0), 3.0 (IQR = 2.0–3.0), 4.0 (IQR = 1.0–4.0), with a total score of 7.0 (IQR = 6.0–9.0). Accordingly, the number of patients with definite, probable, and possible PNS was 42, 49, and 19, respectively. By contrast, a majority acquired a definite diagnosis from the 2004 criteria (97, 85.8%). Among them, only 42.3% (41/97) patients had a definite diagnosis, as the remaining 40, 14, and 2 were re-diagnosed with probable PNS, possible PNS, and non-PNS according to the 2021 criteria (**Figure 3B**). On the contrary, a patient with GABA_B_R encephalitis and SCLC previously diagnosed with possible PNS fulfilled an updated definite diagnosis.

**Table 2:** Definition and classification of the 2 criteria.

	Neurologic phenotype	Antibody	Cancer	Diagnostic level
**2004 criteria**	Classical (n = 80)	Well characterized (n = 67)	Present (n = 90)	Definite (n = 97)
	Non-classical (n = 33)	Partially characterized (n = 0)	Absent (n = 23)	Possible (n = 16)
		Others or negative (n = 46)		
**2021 criteria**	High-risk (n = 68)	High-risk (n = 69)	Found, consistent with phenotype and (if present) antibody, or not consistent but antigen expression demonstrated (n = 69)	Definite (n = 42)
	Intermediate-risk (n = 45)	Intermediate-risk (n = 20)	Not found (or not consistent) but follow-up < 2 years (n = 26)	Probable (n = 49)
	Defined epidemiologically not associated with cancer (n = 0)	Low-risk or negative (n = 24)	Not found but follow-up ≥ 2 years (n = 27)	Possible (n = 19)
				Non-PNS (n = 3)

PNS, paraneoplastic neurologic syndrome.

### Diagnostic Performance of the 2 Criteria in Different Conditions

In consideration of no gold criteria for the PNS, we compared the diagnostic performances of these 2 criteria with given conditions from clinical practice. Generally, the associated antibodies and cancers might be more significant and have specific indicators for the diagnosis of PNS, yet the neurologic phenotypes were also complicated with various mimics. Hence, we divided the cohort into different groups according to the presence of antibodies and cancers for further analyses.

We listed the manifestations of patients in different conditions in **Table 3**. As expected, compared with patients without antibodies, those with antibodies had concomitant cancers more frequently (p = 0.009). The patients with and without antibodies also had significantly different diagnostic levels by the 2004 and 2021 criteria (p < 0.001). Referring to the PNS-Care Score, the scores of all items showed significance, indicating the great utility of antibodies for diagnosis. Similarly, the type of antibodies was significantly different between patients with and without cancers (p = 0.003). Those with cancers had a higher chance of reaching a definite diagnosis by the 2 criteria (both p < 0.001). Notably, among those previously definite patients with concomitant antibodies or cancers, nearly half no longer fulfilled the definite diagnosis according to the 2021 criteria.

**Table 3:** Comparison of the diagnostic criteria under specific conditions.

	Overall (n = 113)	Antibody		p	Cancer		p
		Presence (n = 90)	Absence (n = 23)		Presence (n = 81)	Absence (n = 32)	
Age at onset, years, median (IQR)	60.0 (53.0–65.0)	60.0 (54.0–65.8)	56.0 (50.5–61.5)	0.080	59.0 (53.0–65.0)	60.0 (52.25–65.0)	0.781
Male	69 (61.1)	51 (56.7)	18 (78.3)	0.098	50 (61.7)	19 (59.4)	0.986
**Clinical phenotype**							
*2004 criteria*							
Classical	80 (70.8)	61 (67.8)	19 (82.6)	0.255	58 (71.6)	22 (68.8)	0.943
Non-classical	33 (29.2)	29 (32.2)	4 (17.4)		23 (28.4)	10 (31.2)	
*2021 criteria*							
High-risk	68 (60.2)	50 (55.6)	18 (78.3)	0.081	50 (61.7)	18 (56.2)	0.747
Intermediate-risk	45 (39.8)	40 (44.4)	5 (21.7)		31 (38.3)	14 (43.8)	
**Antibody**							
*2004 criteria*							
Well-characterized	67 (59.3)	67 (74.4)	0 (0.0)	<0.001	46 (56.8)	21 (65.6)	0.389
Partially characterized	0	0	0		0	0	
Others or negative	46 (40.8)	23 (25.6)	23 (100.0)		35 (43.2)	11 (34.3)	
*2021 criteria*							
High-risk	69 (61.1)	69 (76.7)	0 (0.0)	<0.001	48 (59.3)	21 (65.6)	0.003
Intermediate-risk	20 (17.7)	20 (22.2)	0 (0.0)		10 (12.3)	10 (31.2)	
Low-risk/negative	24 (21.2)	1 (1.1)	23 (100.0)		23 (28.4)	1 (3.1)	
**Cancer**							
*2004 criteria*							
Presence	81 (71.7)	59 (65.6)	22 (95.7)	0.009	81 (100.0)	0 (0.0)	<0.001
Absence	32 (28.3)	31 (34.4)	1 (4.3)		0 (0.0)	32 (100.0)	
*2021 criteria*							
Found, consistent with phenotype and (if present) antibody, or not consistent but antigen expression demonstrated	60 (53.1)	43 (47.8)	17 (73.9)	0.081	60 (74.1)	0 (0.0)	<0.001
Not found/consistent but follow-up < 2 years	26 (23.0)	23 (25.6)	3 (13.0)		7 (8.6)	19 (59.4)	
Not found but follow-up ≥ 2 years	27 (23.9)	24 (26.7)	3 (13.0)		14 (17.3)	13 (40.6)	
**Diagnostic level**							
*2004 criteria*							
Definite	97 (85.8)	78 (86.7)	19 (82.6)	0.870	76 (93.8)	21 (65.6)	<0.001
Possible	16 (14.2)	12 (13.3)	4 (17.4)		5 (6.2)	11 (34.4)	
*2021 criteria*							
Definite	42 (37.2)	42 (46.7)	0 (0.0)	<0.001	42 (51.9)	0 (0.0)	<0.001
Probable	49 (43.4)	32 (35.6)	17 (73.9)		27 (33.3)	22 (68.8)	
Possible	19 (16.8)	16 (17.8)	3 (13.0)		9 (11.1)	10 (31.2)	
Non-PNS	3 (2.7)	0 (0.0)	3 (13.0)		3 (3.7)	0 (0.0)	
**PNS-Care Score, median (IQR)**							
Clinical level	3.0 (2.0–3.0)	3.0 (2.0–3.0)	3.0 (3.0–3.0)	0.048	3.0 (2.0–3.0)	3.0 (2.0–3.0)	0.594
Laboratory level	3.0 (2.0–3.0)	3.0 (3.0–3.0)	0.0 (0.0–0.0)	<0.001	3.0 (0.0–3.0)	3.0 (2.0–3.0)	0.159
Cancer	4.0 (1.0–4.0)	1.0 (0.0–4.0)	4.0 (2.50–4.0)	0.033	4.0 (1.0–4.0)	1.0 (0.0–1.0)	<0.001
Total	7.0 (6.0–9.0)	7.0 (6.0–9.0)	7.0 (5.0–7.0)	0.022	8.0 (6.0–10.0)	6.0 (5.0–6.0)	<0.001

Numbers (%) are for all patients unless otherwise stated.

IQR, interquartile range; PNS, paraneoplastic neurologic syndrome.

We then analyzed the diagnostic value of these 2 aspects for the definite PNS diagnosed by the 2 criteria. As shown in **Table 4**, the sensitivity of the criteria represented as the rate of the definite diagnosis among patients with antibody/cancer was inversely proportional to the omission diagnostic rate. And the specificity represented as the rate of non-definite diagnosis among patients without antibody/cancer suggested the degree of misdiagnosis. Referring to the diagnostic performance of each parameter (antibody and cancer), cancer showed better sensitivity and specificity in both criteria. The presence of cancers might play a more important role in the final diagnosis than antibodies. Yet they performed excellently in the specificity of the 2021 criteria in spite of their general sensitivity.

**Table 4:** Diagnostic performance of antibody and cancer for a definite diagnosis of PNS by the 2 criteria.

		Sensitivity	Specificity	Positive Predictive Value	Negative Predictive Value	Accuracy
2004 criteria	Antibody	86.7 (77.9–92.9)	17.4 (5.0–38.8)	80.4 (77.0–83.4)	25.0 (10.6–48.4)	72.6 (63.4–80.5)
	Cancer	93.8 (86.2–98.0)	34.4 (18.6–53.2)	78.4 (73.7–82.4)	68.8 (45.4–85.4)	77.0 (68.1–84.4)
2021 criteria	Antibody	46.7 (36.1–57.5)	100.0 (85.2–100.0)	100.0 (90.0–100.0)	32.4 (28.3–36.8)	57.5 (47.9–66.8)
	Cancer	51.9 (40.5–63.1)	100.0 (89.1–100.0)	100.0 (90.0–100.0)	45.1 (39.6–50.7)	65.5 (56.0–74.2)

All data are stated as percentage with 95% CI confidence interval in the parentheses.

PNS, paraneoplastic neurologic syndrome.

## Discussion

Our study firstly evaluated and compared the diagnostic performance of the 2004 PNS criteria and the 2021 updated revision in a Chinese population. In comparison with the previous criteria, the updated criteria showed a drop of 48.7% in the definite diagnosis rate, with a large proportion (43.4%) turning into probable diagnosis, and 2.7% into the “non-PNS” group. The new PNS-Care Score provided a specific and strict approach to improve the diagnostic accuracy, but its increased risk of underdiagnosis were also needed to be of concern. Above all, the performance of the 2021 criteria could be summarized as follows.

On the one hand, the 2021 criteria increased the accuracy by a strict scoring system in the definite diagnosis. These could be supported by the individual diagnostic performance of the 2 criteria, as a result of an excellent specificity but a lower sensitivity in the 2021 criteria. Referring to the clinical phenotypes, their definitions of terminology were updated and modified according to the latest criteria for a more appropriate summary and classification. For example, the criteria of LE were updated in 2016 (46), yet 12 of 35 patients previously diagnosed with LE no longer met the new criteria and were excluded from the high-risk phenotype.

Moreover, the presence of specific neural antibodies and/or cancer was emphasized in the updated criteria for an unambiguous diagnostic certainty. For instance, among 67 patients diagnosed with definite PNS by the 2004 criteria with well-characterized antibodies and no cancer, there were only 32 (47.8%) meeting the definite level by the 2021 criteria, as the remaining 24 (35.8%) and 11 (16.4%) were probable and possible levels. This might be mainly explained by the larger diagnostic value of cancer itself rather than the antibody in each patient. Meanwhile, among 17 patients with no detectable neural antibodies during follow-up, the diagnosis in 14 patients with a previous definite diagnosis changed into probable (n = 11) and possible (n = 1) diagnoses, as 2 were excluded as non-PNS (each with mucosa-associated lymphoid tissue lymphoma and colorectal carcinoma). Similarly, another patient with inconsistent renal carcinoma was diagnosed with non-PNS. Thus, the presence of associated antibodies and their consistent cancer might be essential for a definite diagnosis. Additionally, the updated criteria could reduce the risk of overdiagnosis and the real burden of PNSs. Nonetheless, we should acknowledge that comprehensive screening for cancers or antibodies was difficult to achieve in all patients, due to their poor economic and physical conditions. Notably, in the case of inconsistent cancers, the demonstration of antigen expression by the tumor was reasonable and was required for the diagnosis. Hence, special attention should be paid to those patients to avoid overdiagnosis, and additional diagnostic evidence including available clinical features and objective ancillary evidence should be further pursued.

On the other hand, the updated criteria provided a broad spectrum of neural antibodies. For example, the anti-SOX1 antibody found in 2005 (47) was newly classified as high-risk antibodies in the 2021 criteria (10). And some specific antibodies against neuronal surface antigens were also included in the specific risk levels according to their frequencies of cancer association regardless of their eventual pathogenic effects (10). As a result, the undefined antibodies in 23 patients by the 2004 criteria were redefined as 2 in high risk (anti-SOX1 antibody), 20 in intermediate risk (anti-GABA_B_R, anti-AMPAR, anti-NMDAR, and anti-VGCC antibodies), and 1 in low risk (anti-AQP4). Among them, the diagnostic levels of 10 patients were changed, including 2 with a previous definite diagnosis changed into probable (1 patient presented with LE with anti-NMDAR antibody and granular cell carcinoma of kidney and followed up for <2 years) and possible (1 patient failed to satisfy the previous definition of LE with anti-GABA_B_R antibody and thymoma and followed up for >2 years). Apart from 7 of the remaining 8 patients with a possible diagnosis that became probable by the 2021 criteria, 1 patient presenting with encephalitis with anti-GABA_B_R antibody and SCLC was diagnosed with definite PNS. Above all, with highlights of a causal association with cancer, the new risk-dependent definition of antibodies facilitated the consideration of probable PNS and might promote an active search for potential cancers.

It is important to note that the 2021 criteria may underestimate the occurrence of PNS without neural antibodies and do not identify it as definite PNS associated with cancer and novel antibodies (the level of risk has not been determined) or low-risk antibodies, even if tumor cells express the neural antigen recognized by the antibody (10). Meanwhile, the lower sensitivity caused by the rigorous criteria might decrease the diagnostic rate, resulting in more patients with a probable/possible diagnosis rather than a definite one. As a result, it might delay appropriate interventions or deprive patients of proper interventions or treatments, further causing irreparable harm. Hence, timely aggressive treatments might be necessary for those with possible or probable diagnoses after ruling out other diseases. Considering that the diagnostic level might change with the discovery of new evidence over time, close monitoring with regular re-examinations was needed. Otherwise, the risk classifications of clinical phenotypes and antibodies might change by the increase of reports and the deepening of understanding and further prompt the continuous revision and update of the diagnostic criteria.

We should acknowledge the limitations of our study. Firstly, as a retrospective study, publication and selection bias is inevitable. Secondly, the diagnoses of these published data were based on the time of publishing, and their further prognoses were not available. Thirdly, the profile of our cohort was partially representative of the Chinese population. In reality, there might be a certain number of PNS patients being underreported. Moreover, the diagnostic performance of the updated criteria should be further evaluated and verified in view of no gold criteria. Thus, prospective larger studies over the world with more complicated combinations with various clinical phenotypes, antibodies, and cancers should be performed for further validation.

## Conclusion

The 2021 PNS criteria provided a better clinical application for avoiding overdiagnosis or misdiagnosis than the 2004 criteria. Indeed, the presence of cancer played a more critical and important role in the definite diagnosis. Comprehensive and long-term screening for cancers is essential in suspected PNS. Above all, the updated PNS criteria showed an accurate diagnostic performance by a comprehensive synthesis of clinical phenotypes, antibodies, and cancers. It would provide the clinical utility with the risk stratification to expedite the diagnostic process by prompting recognition and treatment. Nevertheless, in view of the possible delay or underdiagnosis of PNS induced by the updated criteria, timely treatments and close monitoring with long-term follow-up should be performed to improve the prognosis for those with probable/possible diagnoses.

## Data Availability Statement

The original contributions presented in the study are included in the article/**Supplementary Material**. Further inquiries can be directed to the corresponding authors.

## Ethics Statement

The studies involving human participants were reviewed and approved by the ethics committee of the Second Affiliated Hospital School of Medicine Zhejiang University. Written informed consent for participation was not required for this study in accordance with the national legislation and the institutional requirements.

## Author Contributions

M-TC and Y-XZ contributed to the concept and design of the study. All authors contributed to the acquisition and analysis of the data. M-TC and SQ contributed to drafting the initial manuscript. Y-XZ and M-PD contributed to revising the manuscript for intellectual content. All authors read and approved the final version before submission.

## Funding

This study was supported by the National Natural Science Foundation of China (grant number 82071443).

## Conflict of Interest

The authors declare that the research was conducted in the absence of any commercial or financial relationships that could be construed as a potential conflict of interest.

## Publisher’s Note

All claims expressed in this article are solely those of the authors and do not necessarily represent those of their affiliated organizations, or those of the publisher, the editors and the reviewers. Any product that may be evaluated in this article, or claim that may be made by its manufacturer, is not guaranteed or endorsed by the publisher.
