# Supplementary material for: Evaluation of the Updated Diagnostic Criteria for Paraneoplastic Neurologic Syndromes in China
Source: Front Immunol. 2022 Jan 31;13:790400. doi: 10.3389/fimmu.2022.790400 (PMC8841409; doi:10.3389/fimmu.2022.790400)
Supplement: Supplementary file 1 [file Table_1.doc]

Supplementary Table 1 2004 criteria for PNS.

| Definite PNS |
| --- |
| 1. A classical syndrome and cancer that develops within five years of the diagnosis of the neurological disorder. |
| 2. A non-classical syndrome that resolves or significantly improves after cancer treatment without concomitant immunotherapy, provided that the syndrome is not susceptible to spontaneous remission. |
| 3. A non-classical syndrome with onconeural antibodies (well characterized or not) and cancer that develops within five years of the diagnosis of the neurological disorder. |
| 4. A neurological syndrome (classical or not) with well characterized onconeural antibodies (anti-Hu, Yo, CV2, Ri, Ma2, or amphiphysin), and no cancer. |
| Possible PNS |
| 1. A classical syndrome, no onconeural antibodies, no cancer but at high risk to have an underlying tumour. |
| 2. A neurological syndrome (classical or not) with partially characterized onconeural antibodies and no cancer. |
| 3. A non-classical syndrome, no onconeural antibodies, and cancer present within two years of diagnosis. |

Abbreviation: PNS, paraneoplastic neurologic syndrome.

Supplementary Table 2 PNS-Care Score of the 2021 criteria.

|  | Points |
| --- | --- |
| Clinical level |  |
| High-risk phenotypes | 3 |
| Intermediate-risk phenotypes | 2 |
| Defined phenotype epidemiologically not associated with cancer | 0 |
| Laboratory level |  |
| High-risk antibody (>70% cancer association) | 3 |
| Intermediate risk antibody (30%–70%) | 2 |
| Lower risk antibody (<30%) or negative | 0 |
| Cancer |  |
| Found, consistent with phenotype and (if present) antibody, or not consistent but antigen expression demonstrated | 4 |
| Not found (or not consistent) but follow-up <2 y | 1 |
| Not found and follow-up ≥ 2 y | 0 |
| Diagnostic level |  |
| Definite ≥ 8 |  |
| Probable 6–7 |  |
| Possible 4–5 |  |
| Non-PNS ≤ 3 |  |

Abbreviation: PNS, paraneoplastic neurologic syndrome.

Supplementary Table 3 Characteristics of patients with different antibodies.

|  | Hu  (n=32) | Yo  (n=13) | CV2 (n=10) | Ma2 (n=9) | Amphiphysin (n=5) | SOX1 (n=3) | Ri  (n=2) | GABA_B_R (n=22) | NMDAR (n=4) | AMPAR (n=2) | VGCC (n=2) | AQP4  (n=1) |
| --- | --- | --- | --- | --- | --- | --- | --- | --- | --- | --- | --- | --- |
| Age at onset, years, median [IQR] | 61.0 [58.0-67.5] | 65.0 [61.0-68.0] | 57.5 [54.0-66.5] | 53.0 [47.0-64.0] | 55.0 [52.0-63.0] | 69.0 [65.0-72.0] | 57.5 [51.8-63.3] | 57.5 [53.3-60.8] | 55.5 [51.8-57.5] | 53.0 [51.5-54.5] | 62.5 [61.3-63.8] | 60.0 [-] |
| Males | 19 (59.4) | 5 (38.5) | 6 (60.0) | 4 (44.4) | 1 (20.0) | 3 (100.0) | 1 (50.0) | 15 (68.2) | 3 (75.0) | 1 (50.0) | 1 (50.0) | 0 (0.0） |
| Clinical phenotypes | | | | | | | | | | | | |
| EM | 6 (18.8) | 0 (0.0) | 0 (0.0) | 0 (0.0) | 2 (40.0) | 0 (0.0) | 0 (0.0) | 2 (9.1) | 0 (0.0) | 0 (0.0) | 0 (0.0) | 0 (0.0) |
| LE* | 3 (9.4) | 2 (15.4) | 1 (10.0) | 3 (33.3) | 2 (40.0) | 1 (33.3) | 0 (0.0) | 8 (36.4) | 2 (50.0) | 2 (100.0) | 0 (0.0) | 0 (0.0) |
| SCD / RPCS | 3 (9.4) | 7 (53.8) | 3 (30.0) | 0 (0.0) | 0 (0.0) | 0 (0.0) | 1 (50.0) | 0 (0.0) | 1 (25.0) | 0 (0.0) | 0 (0.0) | 0 (0.0) |
| SNN | 3 (9.4) | 0 (0.0) | 0 (0.0) | 0 (0.0) | 0 (0.0) | 0 (0.0) | 0 (0.0) | 0 (0.0) | 0 (0.0) | 0 (0.0) | 0 (0.0) | 0 (0.0) |
| CGPO | 0 (0.0) | 0 (0.0) | 1 (10.0) | 0 (0.0) | 0 (0.0) | 0 (0.0) | 0 (0.0) | 0 (0.0) | 0 (0.0) | 0 (0.0) | 0 (0.0) | 0 (0.0) |
| LEMS | 2 (6.2) | 1 (7.7) | 0 (0.0) | 0 (0.0) | 0 (0.0) | 2 (66.7) | 0 (0.0) | 0 (0.0) | 0 (0.0) | 0 (0.0) | 2 (100.0) | 0 (0.0) |
| Brainstem encephalitis | 2 (6.2) | 0 (0.0) | 0 (0.0) | 0 (0.0) | 0 (0.0) | 0 (0.0) | 0 (0.0) | 0 (0.0) | 0 (0.0) | 0 (0.0) | 0 (0.0) | 0 (0.0) |
| Encephalitis | 6 (18.8) | 0 (0.0) | 0 (0.0) | 0 (0.0) | 0 (0.0) | 0 (0.0) | 0 (0.0) | 12 (54.5) | 1 (25.0) | 0 (0.0) | 0 (0.0) | 1 (100.0) |
| Motor neuron disease | 0 (0.0) | 1 (7.7) | 0 (0.0) | 1 (11.1) | 0 (0.0) | 0 (0.0) | 0 (0.0) | 0 (0.0) | 0 (0.0) | 0 (0.0) | 0 (0.0) | 0 (0.0) |
| Myelopathy | 1 (3.1) | 1 (7.7) | 1 (10.0) | 0 (0.0) | 0 (0.0) | 0 (0.0) | 1 (50.0) | 0 (0.0) | 0 (0.0) | 0 (0.0) | 0 (0.0) | 0 (0.0) |
| Parkinson syndrome | 0 (0.0) | 0 (0.0) | 1 (10.0) | 0 (0.0) | 0 (0.0) | 0 (0.0) | 0 (0.0) | 0 (0.0) | 0 (0.0) | 0 (0.0) | 0 (0.0) | 0 (0.0) |
| Peripheral neuropathy | 6 (18.8) | 1 (7.7) | 3 (30.0) | 5 (55.6) | 1 (20.0) | 0 (0.0) | 0 (0.0) | 0 (0.0) | 0 (0.0) | 0 (0.0) | 0 (0.0) | 0 (0.0) |
| Detected cancers | 23 (71.9) | 7 (53.8) | 8 (80.0) | 5 (55.6) | 4 (80.0) | 3 (100.0) | 1 (50.0) | 12 (54.5) | 3 (75.0) | 2 (100.0) | 2 (100.0) | 1 (100.0) |
| Cancers | | | | | | | | | | | | |
| SCLC | 8 (25.0) | 1 (7.7) | 3 (30.0) | 0 (0.0) | 2 (40.0) | 3 (100.0) | 0 (0.0) | 5 (22.7) | 1 (25.0) | 1 (50.0) | 1 (50.0) | 0 (0.0) |
| NSCLC | 12 (37.5) | 2 (15.4) | 1 (10.0) | 0 (0.0) | 0 (0.0) | 0 (0.0) | 0 (0.0) | 6 (27.3) | 1 (25.0) | 0 (0.0) | 1 (50.0) | 0 (0.0) |
| Breast carcinoma | 1 (3.1) | 1 (7.7) | 1 (10.0) | 0 (0.0) | 1 (20.0) | 0 (0.0) | 1 (50.0) | 0 (0.0) | 0 (0.0) | 0 (0.0) | 0 (0.0) | 1 (100.0) |
| Thymoma | 0 (0.0) | 0 (0.0) | 0 (0.0) | 1 (11.1) | 0 (0.0) | 0 (0.0) | 0 (0.0) | 1 (4.5) | 0 (0.0) | 1 (50.0) | 0 (0.0) | 0 (0.0) |
| Intestinal cancer | 1 (3.1) | 1 (7.7) | 0 (0.0) | 1 (11.1) | 0 (0.0) | 0 (0.0) | 0 (0.0) | 0 (0.0) | 0 (0.0) | 0 (0.0) | 0 (0.0) | 0 (0.0) |
| Testicular cancer | 0 (0.0) | 0 (0.0) | 0 (0.0) | 1 (11.1) | 0 (0.0) | 0 (0.0) | 0 (0.0) | 0 (0.0) | 0 (0.0) | 0 (0.0) | 0 (0.0) | 0 (0.0) |
| Cervical carcinoma | 0 (0.0) | 0 (0.0) | 1 (10.0) | 0 (0.0) | 0 (0.0) | 0 (0.0) | 0 (0.0) | 0 (0.0) | 0 (0.0) | 0 (0.0) | 0 (0.0) | 0 (0.0) |
| Spinal cord tumor | 0 (0.0) | 0 (0.0) | 0 (0.0) | 0 (0.0) | 1 (20.0) | 0 (0.0) | 0 (0.0) | 0 (0.0) | 0 (0.0) | 0 (0.0) | 0 (0.0) | 0 (0.0) |
| Palatal squamous epithelial carcinoma | 1 (3.1) | 0 (0.0) | 0 (0.0) | 0 (0.0) | 0 (0.0) | 0 (0.0) | 0 (0.0) | 0 (0.0) | 0 (0.0) | 0 (0.0) | 0 (0.0) | 0 (0.0) |
| Ovarian carcinoma | 0 (0.0) | 1 (7.7) | 0 (0.0) | 0 (0.0) | 0 (0.0) | 0 (0.0) | 0 (0.0) | 0 (0.0) | 0 (0.0) | 0 (0.0) | 0 (0.0) | 0 (0.0) |
| Lymphoepithelial carcinona | 0 (0.0) | 1 (7.7) | 0 (0.0) | 0 (0.0) | 0 (0.0) | 0 (0.0) | 0 (0.0) | 0 (0.0) | 0 (0.0) | 0 (0.0) | 0 (0.0) | 0 (0.0) |
| Multiple myeloma | 0 (0.0) | 0 (0.0) | 0 (0.0) | 1 (11.1) | 0 (0.0) | 0 (0.0) | 0 (0.0) | 0 (0.0) | 0 (0.0) | 0 (0.0) | 0 (0.0) | 0 (0.0) |
| Non-hodgkin lymphoma | 0 (0.0) | 0 (0.0) | 0 (0.0) | 1 (11.1) | 0 (0.0) | 0 (0.0) | 0 (0.0) | 0 (0.0) | 0 (0.0) | 0 (0.0) | 0 (0.0) | 0 (0.0) |
| Renal cancer | 0 (0.0) | 0 (0.0) | 1 (10.0) | 0 (0.0) | 0 (0.0) | 0 (0.0) | 0 (0.0) | 0 (0.0) | 1 (25.0) | 0 (0.0) | 0 (0.0) | 0 (0.0) |
| Gastric carcinoma | 0 (0.0) | 0 (0.0) | 1 (10.0) | 0 (0.0) | 0 (0.0) | 0 (0.0) | 0 (0.0) | 0 (0.0) | 0 (0.0) | 0 (0.0) | 0 (0.0) | 0 (0.0) |
| Outcomes | | | | | | | | | | | | |
| Death | 13 (40.6) | 2 (15.4) | 3 (30.0) | 3 (33.3) | 1 (20.0) | 2 (66.7) | 1 (50.0) | 7 (31.8) | 2 (50.0) | 1 (50.0) | 0 (0.0) | 0 (0.0) |
| Aggravation | 3 (9.4) | 2 (15.4) | 2 (20.0) | 0 (0.0) | 1 (20.0) | 0 (0.0) | 1 (50.0) | 1 (4.5) | 0 (0.0) | 0 (0.0) | 0 (0.0) | 0 (0.0) |
| Stability | 7 (21.9) | 1 (7.7) | 0 (0.0) | 2 (22.2) | 2 (40.0) | 0 (0.0) | 0 (0.0) | 0 (0.0) | 0 (0.0) | 0 (0.0) | 1 (50.0) | 0 (0.0) |
| Remission | 9 (28.1) | 8 (61.5) | 5 (50.0) | 3 (33.3) | 0 (0.0) | 1 (33.3) | 0 (0.0) | 14 (63.6) | 2 (50.0) | 1 (50.0) | 1 (50.0) | 1 (100.0) |
| Diagnostic levels by 2004 criteria | | | | | | | | | | | | |
| Definite | 32 (100.0) | 13 (100.0) | 10 (100.0) | 9 (100.0) | 5 (100.0) | 3 (100.0) | 2 (100.0) | 12 (54.5) | 3 (75.0) | 2 (100.0) | 2 (100.0) | 0 (0.0) |
| Possible | 0 (0.0) | 0 (0.0) | 0 (0.0) | 0 (0.0) | 0 (0.0) | 0 (0.0) | 0 (0.0) | 10 (45.5) | 1 (25.0) | 0 (0.0) | 0 (0.0) | 1 (100.0) |
| Diagnostic levels by 2021 criteria | | | | | | | | | | | | |
| Definite | 20 (62.5) | 5 (38.5) | 4 (40.0) | 1 (11.1) | 3 (60.0) | 3 (100.0) | 1 (50.0) | 11 (50.0) | 2 (50.0) | 2 (100.0) | 2 (100.0) | 0 (0.0) |
| Probable | 10 (31.2) | 6 (46.2) | 5 (50.0) | 3 (33.3) | 1 (20.0) | 0 (0.0) | 1 (50.0) | 6 (27.3) | 2 (50.0) | 0 (0.0) | 0 (0.0) | 1 (100.0) |
| Possible | 2 (6.2) | 2 (15.4) | 1 (10.0) | 5 (55.6) | 1 (20.0) | 0 (0.0) | 0 (0.0) | 5 (22.7) | 0 (0.0) | 0 (0.0) | 0 (0.0) | 0 (0.0) |
| Non-PNS | 0 (0.0) | 0 (0.0) | 0 (0.0) | 0 (0.0) | 0 (0.0) | 0 (0.0) | 0 (0.0) | 0 (0.0) | 0 (0.0) | 0 (0.0) | 0 (0.0) | 0 (0.0) |

Numbers (%) are for all patients unless otherwise stated.

*Diagnosed by 2016 Lancet criteria (Graus F, Titulaer MJ, Balu R, Benseler S, Bien CG, Cellucci T, et al. A clinical approach to diagnosis of autoimmune encephalitis. Lancet Neurol (2016) 15 (4):391-404.).

Abbreviations: AMPAR, α-amino-3-hydroxy-5-methyl-4-isoxazolepropionic acid receptor; AQP4, aquaporin 4; CGPO, chronic gastrointestinal pseudo-obstruction; EM, encephalomyelitis; GABA_B_R, gamma-aminobutyric acid-b receptor; IQR, interquartile range; LE, limbic encephalitis; LEMS, Lambert-Eaton myasthenic syndrome; NMDAR, N-methyl-D-aspartate receptor; NSCLC, non–small-cell lung cancer; PNS, paraneoplastic neurologic syndrome; RPCS, rapidly progressive cerebellar syndrome; SCLC, small-cell lung cancer; SNN, sensory neuronopathy; VGCC, voltage-gated calcium channel.

Supplementary Table 4 Characteristics of patients with specific cancers.

|  | SCLC  (n=32) | NSCLC  (n=18) | Breast carcinoma (n=7) | Ovarian carcinoma (n=2) | Lymphoma (n=1) |
| --- | --- | --- | --- | --- | --- |
| **Age at onset, years, median [IQR]** | 61.0 [55.8-66.3] | 58.50 [56.0-61.0] | 60.0 [49.0-61.50] | 51.0 [44.0-58.0] | 47.0 [47.0-47.0] |
| Males | 26 (81.2) | 12 (66.7) | 0 (0.0) | 0 (0.0) | 1 (100.0) |
| Clinical phenotypes | | | | | |
| EM | 1 (3.1) | 2 (11.1) | 1 (14.3) | 0 (0.0) | 0 (0.0) |
| LE* | 8 (25.0) | 3 (16.7) | 0 (0.0) | 0 (0.0) | 0 (0.0) |
| SCD / RPCS | 3 (9.4) | 1 (5.6) | 1 (14.3) | 2 (100.0) | 0 (0.0) |
| SNN | 2 (6.2) | 0 (0.0) | 0 (0.0) | 0 (0.0) | 0 (0.0) |
| CGPO | 1 (3.1) | 0 (0.0) | 0 (0.0) | 0 (0.0) | 0 (0.0) |
| LEMS | 8 (25.0) | 4 (22.2) | 0 (0.0) | 0 (0.0) | 0 (0.0) |
| Encephalitis | 5 (15.6) | 5 (27.8) | 1 (14.3) | 0 (0.0) | 0 (0.0) |
| Motor neuron disease | 0 (0.0) | 1 (5.6) | 0 (0.0) | 0 (0.0) | 0 (0.0) |
| Myelopathy | 2 (6.2) | 1 (5.6) | 1 (14.3) | 0 (0.0) | 0 (0.0) |
| Parkinson syndrome | 0 (0.0) | 0 (0.0) | 1 (14.3) | 0 (0.0) | 0 (0.0) |
| Peripheral neuropathy | 2 (6.2) | 1 (5.6) | 2 (28.6) | 0 (0.0) | 1 (100.0) |
| Antibodies | | | | | |
| Hu | 8 (25.0) | 12 (66.7) | 1 (14.3) | 0 (0.0) | 0 (0.0) |
| Yo | 1 (3.1) | 2 (11.1) | 1 (14.3) | 1 (50.0) | 0 (0.0) |
| Ri | 0 (0.0) | 0 (0.0) | 1 (14.3) | 0 (0.0) | 0 (0.0) |
| Ma2 | 0 (0.0) | 0 (0.0) | 0 (0.0) | 0 (0.0) | 1 (100.0) |
| Amphiphysin | 2 (6.2) | 0 (0.0) | 1 (14.3) | 0 (0.0) | 0 (0.0) |
| CV2 | 3 (9.4) | 1 (5.6) | 1 (14.3) | 0 (0.0) | 0 (0.0) |
| SOX1 | 3 (9.4) | 0 (0.0) | 0 (0.0) | 0 (0.0) | 0 (0.0) |
| GABA_B_R | 5 (15.6) | 6 (33.3) | 0 (0.0) | 0 (0.0) | 0 (0.0) |
| NMDAR | 1 (3.1) | 1 (5.6) | 0 (0.0) | 0 (0.0) | 0 (0.0) |
| AMPAR | 1 (3.1) | 0 (0.0) | 0 (0.0) | 0 (0.0) | 0 (0.0) |
| VGCC | 1 (3.1) | 1 (5.6) | 0 (0.0) | 0 (0.0) | 0 (0.0) |
| Coexisting antibodies | 3 (9.4) | 8 (44.4) | 0 (0.0) | 0 (0.0) | 0 (0.0) |
| Outcomes |  |  |  |  |  |
| Death | 15 (46.9) | 7 (38.9) | 0 (0.0) | 0 (0.0) | 0 (0.0) |
| Aggravation | 2 (6.2) | 1 (5.6) | 1 (14.3) | 0 (0.0) | 0 (0.0) |
| Stability | 6 (18.7) | 6 (33.4) | 1 (14.3) | 1 (50.0) | 1 (100.0) |
| Remission | 9 (28.1) | 4 (22.2) | 5 (71.4) | 1 (50.0) | 0 (0.0) |
| Diagnostic levels by 2004 criteria | | | | | |
| Definite | 30 (93.8) | 17 (94.4) | 6 (85.7) | 2 (100.0) | 1 (100.0) |
| Possible | 2 (6.2) | 1 (5.6) | 1 (14.3) | 0 (0.0) | 0 (0.0) |
| Diagnostic levels by 2021 criteria | | | | | |
| Definite | 22 (68.8) | 14 (77.8) | 3 (42.9) | 1 (50.0) | 0 (0.0) |
| Probable | 10 (31.2) | 4 (22.2) | 3 (42.9) | 1 (50.0) | 1 (100.0) |
| Possible | 0 (0.0) | 0 (0.0) | 1 (14.3) | 0 (0.0) | 0 (0.0) |

Numbers (%) are for all patients unless otherwise stated.

*Diagnosed by 2016 Lancet criteria (Graus F, Titulaer MJ, Balu R, Benseler S, Bien CG, Cellucci T, et al. A clinical approach to diagnosis of autoimmune encephalitis. Lancet Neurol (2016) 15 (4):391-404.).

Abbreviations: AMPAR, α-amino-3-hydroxy-5-methyl-4-isoxazolepropionic acid receptor; AQP4, aquaporin 4; CGPO, chronic gastrointestinal pseudo-obstruction; EM, encephalomyelitis; GABA_B_R, gamma-aminobutyric acid-b receptor; IQR, interquartile range; LE, limbic encephalitis; LEMS, Lambert-Eaton myasthenic syndrome; NMDAR, N-methyl-D-aspartate receptor; NSCLC, non–small-cell lung cancer; PNS, paraneoplastic neurologic syndrome; RPCS, rapidly progressive cerebellar syndrome; SCLC, small-cell lung cancer; SNN, sensory neuronopathy; VGCC, voltage-gated calcium channel.
